# Digital orthopedics in the new AI era: from ASIA aspect

**DOI:** 10.1186/s42836-023-00220-4

**Published:** 2023-12-13

**Authors:** Yan Wang

**Affiliations:** Department of Orthopaedics, General Hospital of Chinese People’s Liberation Army, Beijing, 100853 China

**Keywords:** Artificial intelligence, Orthopedics, Robotics, Medical education

## Abstract

This editorial explores the transformative impact of artificial intelligence (AI) on orthopedics, with a particular focus on advancements in Asia. It delves into the integration of AI in hospitals, advanced applications in China, and future expectations. The discussion is underpinned by an examination of AI's role in assisted diagnosis, treatment planning, surgical navigation, predictive analysis, and post-operative rehabilitation monitoring.

## Introduction

Artificial intelligence (AI) is the ability of machines or systems to perform tasks that normally require human intelligence, such as reasoning, learning, decision-making, and problem-solving. AI has been developing rapidly in recent years, thanks to the advances in computing power, data availability, and algorithm design. AI has also been transforming many industries, such as manufacturing, transportation, education, entertainment, and health care.

The dawn of the AI era brought significant investment and rapid advancements in language and visual model integration. This technological evolution has sparked a global wave of innovation, with AI applications increasingly permeating various sectors, including healthcare. A notable example is the development of a robot by the Stanford team in July 2023, which integrates language and visual models to execute commands without specific training [1]. Furthermore, NVIDIA released a system for fine reproduction and localization of human organs [2]. OpenAI also exhibited a robot solving a Rubik’s Cube with a single hand [3]. Those go beyond traditional algorithms and involve the complexities of human touch, movement, and coordination with vision. More recently, Apple Inc. issued a new product, Apple Vision Pro, which might engage the development of using virtual reality/augmented reality in medical professional education [4].

Those advancements are regarded as the most cutting-edge and complex breakthroughs in the field of artificial intelligence, paving the way for a new era of AI applications in various sectors, including healthcare. Those ground-breaking developments, along with others, have opened new possibilities in the medical field.

## Artificial intelligence in orthopedics

AI's role in orthopedics is multifaceted [5], ranging from assisting in diagnosis [6, 7] to aiding in post-operative rehabilitation monitoring [8]. AI-based image data analysis, for instance, has proven highly precise and is now being used in clinical trials for orthopedics [9]. This is a significant step forward, as AI’s ability to analyze and interpret complex medical images allows for more accurate diagnoses, better patient outcomes, and more streamlined healthcare processes [10].

Pre-planning and virtual simulated surgeries are other areas where AI has made significant strides [11, 12]. These advancements allow surgeons to plan and simulate surgeries before they take place, reducing the risk of complications and improving patient outcomes [13]. This is a testament to the transformative power of AI in the field of orthopedics, where precision and accuracy are of paramount importance [14].

Robot-assisted surgery is another area where AI has made significant strides [15]. It now represents an impressive 12% of all joint replacement procedures performed in the United States [16]. There are several products that have been widely used in joint replacement worldwide, such as Mako, Rosa, Cori, etc. This trend is expected to continue as more hospitals adopt robot-assisted surgery and as the technology continues to improve [17]. The integration of AI in surgical procedures not only enhances precision but also reduces the risk of human error, leading to better patient outcomes.

AI can also help improve medical education for orthopedic surgeons and trainees by providing interactive, adaptive, and personalized learning tools [18]. AI could act as a mentor to provide personalized advice and support to learners throughout their orthopedic training and career. AI simulators can also provide feedback and guidance to learners during the simulation and measure their outcomes and competencies.

Asia is one of the fastest-growing regions in the world in terms of population, economy, and technology. Asia is also facing many challenges in terms of health care, such as an aging population, increasing chronic diseases, and limited resources. AI and orthopedic surgical robots, e.g., TINAVI, KUNWU, etc., have great potential to address some of these challenges by improving the quality, efficiency, and accessibility of orthopedic services in China [19].

In partnership with clinical institutes and a high-tech company, ALLinMD, an orthopedic medical platform, developed an intelligent assisted diagnosis system for orthopedic outpatient medical records [20]. This system uses AI to analyze and interpret medical records, aiding doctors in diagnosis and treatment planning. This not only improves the accuracy of diagnosis but also reduces the workload for medical professionals, allowing them to focus on more critical aspects of patient care.

**Fig. 1:**
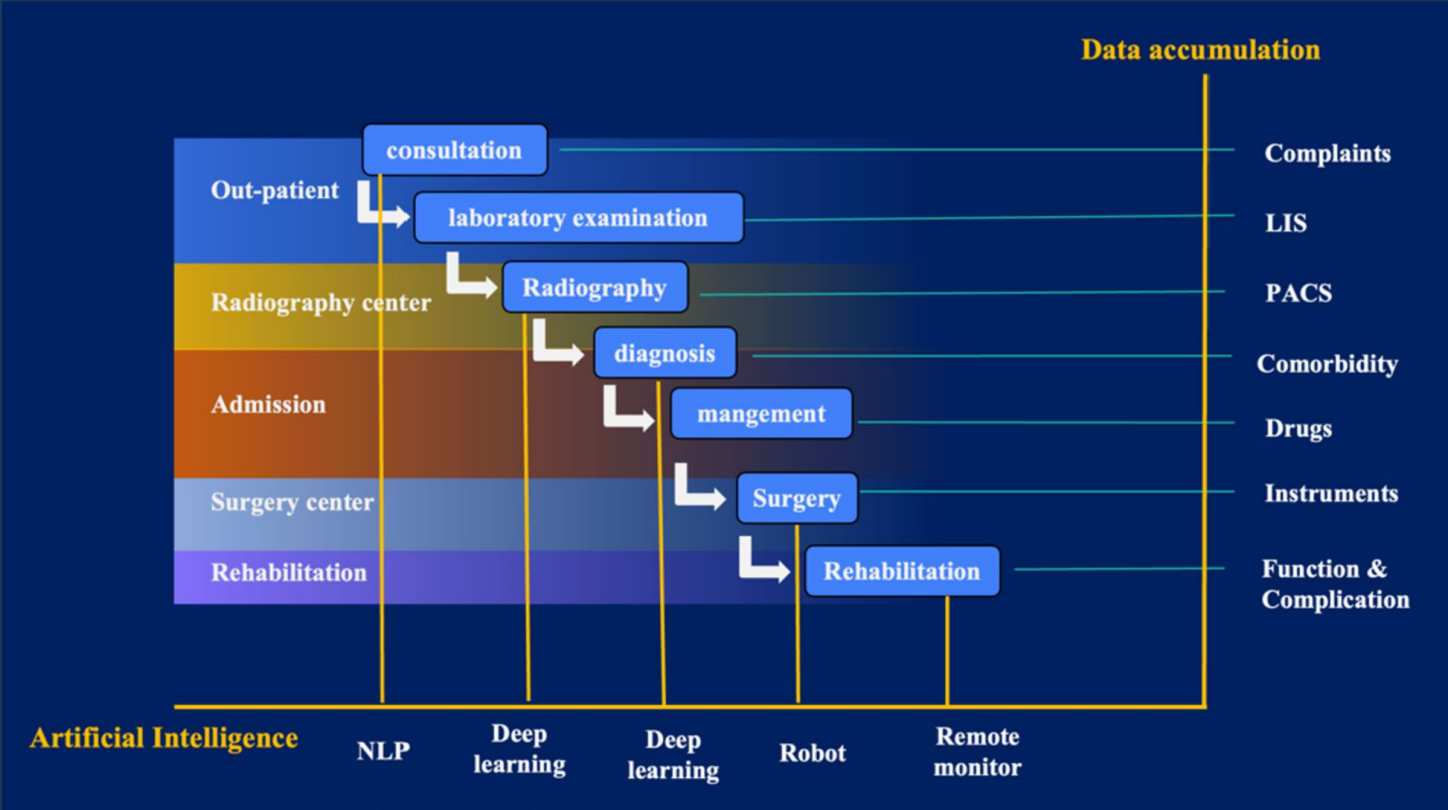
Accumulation of comprehensive clinical data

China has also seen the development of a real-world data platform for orthopedic surgery. This system assists in planning patients’ routine treatment and rehabilitation pathways (Fig. 1) and facilitates the seamless registration of patient information, which is crucial for advancing China’s joint registry system. It will be another exciting improvement reported by Microsoft and Google recently that AI could power those real-world data to be integrated analyzed and exhibited as dashboards, which allow surgeons to be alerted for any potential during the treatment [21, 22].

Moreover, the platform developed by ALLinMD allows for the automatic generation of standardized medical records, which greatly improves data quality and usability [20]. This is a significant advancement, as electronic medical records have traditionally been time-consuming and difficult to standardize, making subsequent analysis challenging. With this platform, doctors can perform preliminary data analysis in real-time, exploring and validating new ideas as they emerge.

Recent research from NYU Langone Hospital, which used more than five million clinical records to train a specialized language model, provides a glimpse into the future of AI in orthopedics [23]. The anticipation is for a universal orthopedic medical language model, fine-tuned with data from local hospitals to align more closely with local healthcare practices.

The value of digital orthopedics lies in its precision, evidence-based approach, efficiency, and affordability. The future trend of digital orthopedics will likely involve the establishment of specialized data platforms that integrate multiple sources and cover the entire medical process. This approach will improve the consistency of orthopedic diagnostic treatment and education, thereby raising the overall standard of orthopedic healthcare.

The application of artificial intelligence (AI) in orthopedics holds immense potential, but it also presents several risks and challenges. (1) One significant concern is the accuracy of AI algorithms. While AI can analyze vast amounts of data quickly, its predictions and analyses can be misleading or incorrect, potentially leading to misdiagnoses or inappropriate treatment plans; (2) Another challenge is the ethical implications of AI use. AI could inadvertently breach patient privacy or confidentiality if not properly managed, and there is also the question of who bears responsibility if an AI-powered system makes a mistake; (3) Additionally, there is the challenge of explainability. AI’s decision-making process can be opaque, often referred to as a “black box”. This lack of transparency can make it difficult for doctors to trust and understand AI’s recommendations, which is crucial in a field where decisions can have life-altering consequences; (4) Lastly, regulatory hurdles pose a significant challenge. The healthcare industry is heavily regulated, and AI applications must comply with these regulations, which may slow down implementation and innovation.

## Conclusion

The development of digital technologies will have a significant impact on orthopedic diagnosis and treatment in the near future. Healthcare professionals need to learn and have a quick knowledge of how to use AI-assisted techniques in daily medical practice. While AI has the potential to revolutionize orthopedics, these risks and challenges must be addressed to ensure safe, ethical, and effective use.
